# Acylation of Chiral Alcohols: A Simple Procedure for Chiral GC Analysis

**DOI:** 10.1155/2012/452949

**Published:** 2012-05-09

**Authors:** Mireia Oromí-Farrús, Mercè Torres, Ramon Canela

**Affiliations:** ^1^Chemistry Department, University of Lleida, 25198 Lleida, Spain; ^2^Food Technology Department, University of Lleida, 25198 Lleida, Spain

## Abstract

The use of iodine as a catalyst and either acetic or trifluoroacetic acid as a derivatizing reagent for determining the enantiomeric composition of acyclic and cyclic aliphatic chiral alcohols was investigated. Optimal conditions were selected according to the molar ratio of alcohol to acid, the reaction time, and the reaction temperature. Afterwards, chiral stability of chiral carbons was studied. Although no isomerization was observed when acetic acid was used, partial isomerization was detected with the trifluoroacetic acid. A series of chiral alcohols of a widely varying structural type were then derivatized with acetic acid using the optimal conditions. The resolution of the enantiomeric esters and the free chiral alcohols was measured using a capillary gas chromatograph equipped with a CP Chirasil-DEX CB column. The best resolutions were obtained with 2-pentyl acetates (*α* = 3.00) and 2-hexyl acetates (*α* = 1.95). This method provides a very simple and efficient experimental workup procedure for analyzing chiral alcohols by chiral-phase GC.

## 1. Introduction

Chiral alcohols occur as natural products and frequently as intermediates in the synthesis of chiral molecules, most of them in the field of synthetic pharmaceuticals possessing chiral centres [1–3]. In pharmacy the use of enantiopure new drugs will certainly increase due to the often well-documented different biological activities of enantiomers. Moreover, the pharmacokinetics or toxicology of each enantiomer with regard to the drug dosage or side effects is significantly different and consequently so are the resulting regulatory requirements [4, 5]. The determination of the enantiomeric excess (% ee) is therefore critical to the progress of these fields, so many methods have been developed for determining the degree of enantiomeric purity of chiral alcohols in the yield of chromatography and electrophoresis [6–12]. Nowadays, gas-liquid chromatography on chiral stationary phases, especially per-0-modified cyclodextrins, plays the dominant role for the chiral separation of a wide range of volatile compounds due to its ease of use and the commercial availability of columns [13]. However, many of these methods for determining the degree of enantiomeric purity of chiral alcohols are improved when these compounds are converted into volatile esters, such as acetate or trifluoroacetate. Acylation reduces polarity and enhances the separation of chiral compounds in the chromatographic column, as well as conferring better volatility. Typically, only derivatization with acetyl groups or with fluorinated acyl groups up to heptafluorobutyryl improves volatility [14].

Acylation of alcohols is among the most frequently used processes in organic synthesis. Although different methods are described in the literature [15–20], some of them are less effective or ineffective for secondary and tertiary alcohols, others are moisture sensitive or highly expensive, and they may even be potentially explosive (e.g., perchlorates or perchloric acid).

Various acylation reactions using iodine as catalyst have been reported [21–26]. Ramalinga and coworkers described iodine as a Lewis catalyst for the esterification and transesterification of acids using an excess of alcohol under reflux conditions [27]. Chavan and coworkers described the transesterification in toluene of *β*-ketoesters with some primary, secondary alcohols and phenols using iodine as a catalyser in the presence of zinc as a mediator [28]. Afterwards, they described that even iodine acts as an efficient catalysts for transesterification reactions; however, phenols did not undergo transesterification [29]. A procedure for the acetylation of alcohols, amines and phenols with isopropenyl acetate and iodine as a catalyser under solvent-free conditions were described by Ahmed and van Lier [30]. This procedure gave acetone as a by-product.

Recently, Jereb and coworkers have demonstrated that iodine is an efficient catalyst for esterification under solvent-free conditions for several alcohols [31].

**Scheme 1:**
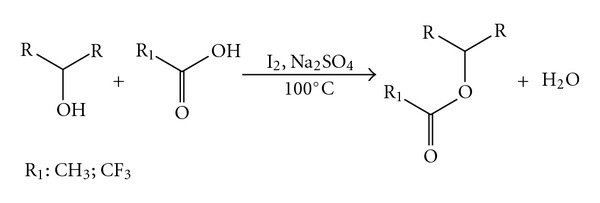
Acylation reaction.

We describe herein a simple and convenient procedure for acylation of chiral alcohols under solvent-free conditions in the presence of a catalytic amount of iodine with no by-products formation and using near equimolar amounts of alcohol and carboxylic acid (Scheme 1). Initially, a systematic study was carried out for catalytic evaluation of iodine in the acetylation of 2-heptanol. Further, the optimized method was applied to (*R*)-2-heptanol and *cis*-1,3-cyclohexanediol to determine that no isomerization occurred with acetylation or trifluoroacetylation. Finally, it was applied to a variety of chiral alcohols. All the esters were analyzed by gas chromatography on a CP Chirasil-DEX CB column in order to provide optimum resolution for a chiral alcohol of a particular structural type.

## 2. Experimental

### 2.1. Reagents

3-Hexanol (4), 4-methyl-2-pentanol (5), 3-methylcyclopentanol (9), 3-methylcyclohexanol (10), 2-*tert*-butylcyclohexanol (11), 2-methylcyclopentanol (12), 4-methylcyclohexanol (13), 2-chlorocyclohexanol (14), 2,6-dimethylcyclohexanol (15), 4*-tert*-butylcyclohexanol (16), 3,3,5-trimethylcyclohexanol (17), 2-phenylcyclohexanol (18), *DL*-menthol (19), 1,2-cyclohexanediol (20), 1,3-cyclohexanediol (21), iodine, and acetic acid were all from Acros Organics, Barcelona, Spain. 2-Butanol (1), *S*-2-butanol, 2-hexanol (3), *S*-2-hexanol, *trans*-*S*,*S*-1,2-cyclohexanediol, *trans*-*R*,*R*-1,2-cyclohexanediol, 2-heptanol (6), *R*-2-heptanol, (+)-menthol, and *tert*-butanol were purchased from Fluka, Madrid, Spain. 2-Octanol (7), 3-octanol (8), and *cis*-1,2-cyclohexanediol were from Sigma-Aldrich, Madrid, Spain. 2-Pentanol (2) was acquired from Merck, Barcelona, Spain. 1-Methylhexyl acetate was obtained by stirring at 100°C for 48 h in a screw-cap vial a mixture of 2-heptanol (20 mmol), acetic acid (200 mmol), iodine (0.6 mmol) and anh. Na_2_SO_4_ (0.2 mmol). Afterwards, 25 mL of hexane were added and the mixture was filtered. The organic solution was washed with saturated sodium thiosulfate solution, saturated NHCO_3_ solution, and water. Hexane was stripped off by distillation at 69°C at atmospheric temperature and the 1-methylhexyl acetate was obtained. The product was characterized by NMR ^1^H and ^13^C.

### 2.2. Procedures

#### 2.2.1. Optimization of the Derivatization Step

Mixtures of 2-heptanol (2 mmol), acetic acid at different molar ratios (2, 3, 4, 12, and 20 mmol) containing iodine (0.06 mmol) and tridecane (4 mmol) as internal standard, anh. Na_2_SO_4_ (0.02 mmol) either without solvent or dissolved in 0.5 mL (7 mmol) *tert*-butanol were stirred at different temperatures (100, 120, and 140°C) for different reaction times (4, 8, 24 and 48 h) in 3 mL amber screw-cap vials. The reaction product was dissolved in 1 mL dichloromethane and filtered. The filtrate was used for GC analysis directly and analysed in a DB-Wax capillary column as described in the following. Reactions were carried out in triplicate.

#### 2.2.2. Derivatization

 A mixture of alcohol (2 mmol), either acetic acid or trifluoroacetic acid (3 mmol, iodine (0.06 mmol), and anh. Na_2_SO_4_ (0.02 mmol)) were stirred at 100°C for 48 h in a 3 mL amber screw-cap vial. The reaction product was dissolved in 1 mL dichloromethane, filtered and analysed by both GC/MS and chiral phase GC analysis as described in what follows. Reactions were carried out in duplicate.

#### 2.2.3. Chromatographic Conditions

**(1)* No Chiral-Phase GC Analysis***

GC-FID analyses were carried out in a Trace 2000 series (ThermoQuest) GC with a DB-Wax (polyethylene glycol) capillary column of 30 m × 0.25 mm diameter, 0.25 *μ*m film thickness. Helium (1 mL/min) was used as the carrier gas. *T*_injector_ =   250°C, *T*_detector_ = 275°C. The GC temperature was programmed at 70°C and ramped first at 5°C/min to 160°C and later at 10°C/min to 200°C.

**(2)* Chiral-Phase GC Analysis***

The column used was CP Chirasil-DEX CB Varian (modified *β*-cyclodextrins bonded to a dimethylpolysiloxane) (25 m × 0.25 mm diameter, 0.25 *μ*m film thickness). Hydrogen (80 cm/s) was used as the carrier gas. *T*_injector_ = 230°C, *T*_detector_ = 250°C. The separation factor, *α*, was calculated according to IUPAC [32].

#### 2.2.4. RMN

^1^H and ^13^C NMR spectra were recorded on a Varian AS400 spectrometer, operating at 400 MHz.

### 2.3. Statistical Analysis

Linear model analysis of variance (ANOVA) and the Tukey-Kramer pairwise differences adjustment method was carried out by the SAS software version 9.0 (SAS Institute, Inc). All the statistical tests applied in this work were employed to determine the statistical differences among 1-methylhexyl acetate yields when reaction optimizations were carried out.

## 3. Results and Discussion

First of all, the acetylation was studied with the presence of a tertiary alcohol, *tert*-butanol, or without solvent, at various molar ratios alcohol : acetic acid with 1 equiv. of acetic acid in the presence of 3 mol % of iodine at 100°C for 24 and 48 h (Table 1). Clearly, *tert*-butanol makes the reaction slower, needing a 1 : 6 molar ratio and 48 h (entry 11). We obtained the maximum yield at a 1 : 2 molar ratio for 24 h without using any solvent (entry 1). Similar behaviour has also been described by Jereb and coworkers [31] using dichloromethane as solvent. They carried out the same reaction with other alcohols using a 1 : 3 molar excess of acetic acid in a free solvent system. The addition of dichloromethane as solvent also provoked a decrease of the reaction rate.

Then, we investigated the influence of lower molar ratios alcohol : acetic acid and different reaction times in a solvent-free system (Table 2). With a 1 : 1 molar ratio alcohol : acid the best yield was obtained after 48 h reaction (entry 2). With 1 : 1.5 and 1 : 2 molar ratios acetylation only required half the time (24 h) to obtain no statistically different results (entries 4, 8). Remarkably in terms of atom economy, a 1 : 1 molar ratio was sufficient, but a longer reaction time was needed to reach a high yield. In order to shorten the reaction times, complementary studies were carried out, increasing the reaction temperature (Table 3). However, the yield of 1-methylhexyl acetate decreased when the temperature increased.

We decided to carry out the subsequent reactions using a 1 : 1.5 molar ratio alcohol : acid at 100°C for 24 h in a solvent-free system. However, considering that the main target was to determine the % ee of a chiral alcohol, it could be not necessary to achieve a high yield of acetylation. Therefore, an equimolecular molar ratio alcohol : acid at 100°C for 4 h could be sufficient. This method is a simple and greener alternative to conventional methods that typically are performed with activated carboxylic acid derivatives such as acid anhydrides, acid chlorides, acyl imidazoles, or acylureas, which need the presence of tertiary amines such as triethylamine, pyridine, or DMAP [15–20]. Moreover, any solvent was needed [28, 29], there were not any byproducts formation [30], and an equimolar amount of reactives can be used [31].

**Figure 1:**
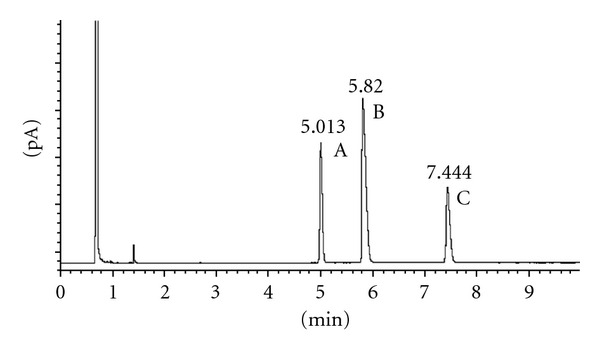
Chiral GC analysis of 2-heptyl acetate. (A) (*S*)-2-heptyl acetate; (B) 2-heptanol; (C) (*R*)-2-heptyl acetate.

**Figure 2:**
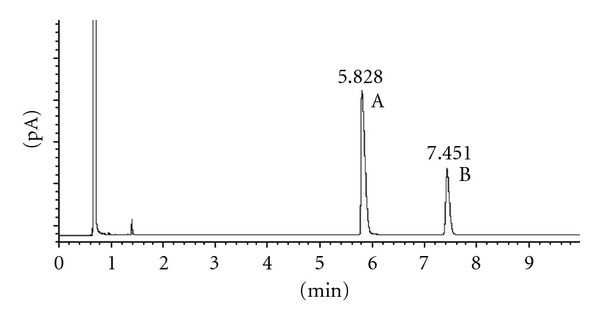
Chiral GC analysis of (*R*)-2-heptyl acetate. (A) (*R*)-2-heptanol; (B) (*R*)-2-heptyl acetate.

**Figure 3:**
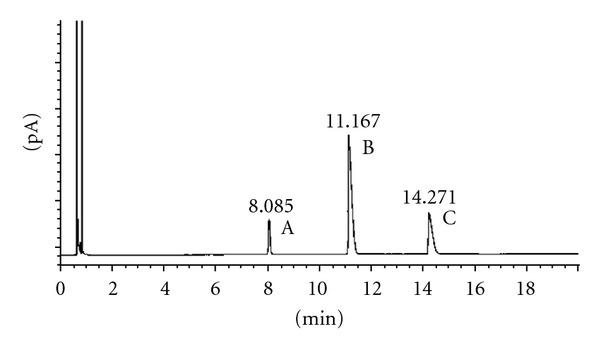
Chiral GC analysis of the *trans*-*S*,*S*-1,2-cyclohexanediol acetates: (A) *trans*-*S*,*S*-1,2-cyclohexanediol diacetate; (B) *trans*-*S*,*S*-2-hydroxycyclohexyl acetate; (C) *trans*-*S*,*S*-1,2-cyclohexanediol.

**Figure 4:**
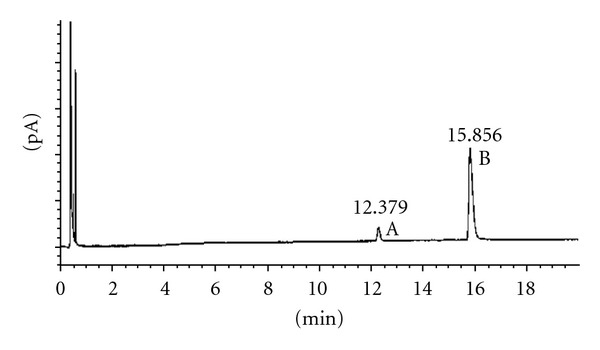
Chiral GC analysis of the *trans*-*R*,*R*-1,2-cyclohexanediol acetates: (A): *trans*-*R*,*R*-2-hydroxycyclohexyl acetate; (B)* trans*-*R*,*R*-1,2-cyclohexanediol.

**Figure 5:**
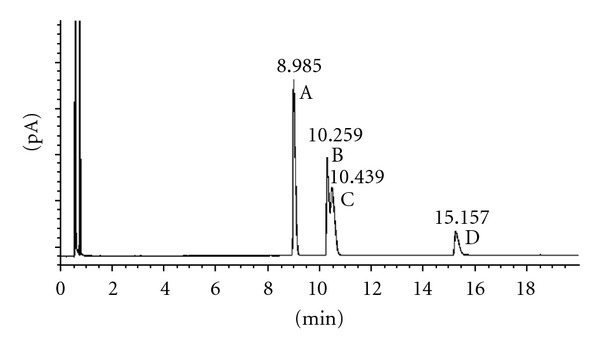
Chiral GC analysis of the *cis*-1,2-cyclohexanediol acetates: (A) *cis*-1,2-cyclohexanediol diacetate; (B) (+/−)-*cis*-2-hydroxycyclohexyl acetate; (C) (+/−)-*cis*-2-hydroxycyclohexyl acetate; (D) *cis*-1,2-cyclohexanediol.

According to these results, we proved the stability of the chiral carbons in the selected conditions. Figures 1 and 2 show the results obtained after acetylation of racemic 2-heptanol and (*R*)-2-heptanol. Similar results were obtained using *trans*-1,3-cyclohexanediol and *cis*-1,3-cyclohexanediol (Figures 3, 4, and 5), indicating that the derivatization process did not cause any isomerization of the chiral carbons of the two molecules. Jereb and coworkers also studied the stereochemical behaviour of some secondary cyclic aliphatic alcohols and 1-phenylethanols in their acetylation reactions with iodine as a catalyst. The aliphatic cyclic alcohols studied yielded esters with retention of stereochemistry, but a loss of stereochemical integrity was observed in chiral 1-phenylethanols. No data was provided about the behaviour of acyclic secondary alcohols or diols in these conditions [31]. Moreover, we also studied the stability of these chiral carbons using trifluoroacetic acid instead of acetic acid. In this case, a partial isomerization was observed making unsuitable this acid for acylation (results not showed). 

Afterwards, three series of alcohols were tested with this acylation method to determine the applicability of the method to study the stereochemical ratio between isomers. The improvement in the separation factors (*α*) related to the free alcohols was determined. A resolution factor of 1.5 or greater indicates baseline enantiomeric resolution [13]. 

Table 4 shows the results when acyclic alcohols were derivatized. Acetylation increased the separation factor of the majority of the tested alcohols. The best *α* was obtained with 2-pentyl acetates that have a separation factor value of 3.00 compared with the 1.07 corresponding to the free alcohols. To our knowledge no better *α* are described in the literature for the 2-pentyl acetates. Higher *α* increases were also observed for 2-butanol acetate derivatives (1.44 compared with 1.05), 2-hexanol acetate derivatives (1.95 compared with 1.05) and 2-octanol acetate derivatives (1.50 compared with 1.02). Acetylation also allowed the two enantiomers of 2-heptanol and 3-octanol to be separated. Smith and Simpson [33] investigated the separation of enantiomers of these acyclic alcohols on a *γ*-trifluoroacetylated cyclodextrin phase at 35°C, showing for the majority of them an *α* value similar to or lower than those obtained in our *β*-cyclodextrin column and lower than our corresponding acylated derivatives. 

The elution order of the acetylated enantiomers of some alcohols, entries 2, 4, 6,7, and 8, was assumed from the literature [34, 35]. We would remark that the elution order of (*−*)-enantiomers and (*+*)-enantiomers depends on some factors such as cyclodextrin type (*α*, *β*, *γ*: on a *β*-CD derivative all (*−*)-enantiomers eluted before the (+)-enantiomers), the stereoconfiguration of the product (it can have a dominant influence on chiral interactions, leading to the reversal of the elution order), cyclodextrin derivative types, and temperature. Moreover, some studies indicate that the retention of enantiomers is correlated with optical activity [36]. 

Table 5 shows the results when mono- and polysubstituted cyclic alcohols were derivatized. Derivatization usually allows to separate the stereoisomers not resolved as free alcohols. Acylation with acetic acid allows the four stereoisomers of 3-methyl-1-cyclopentanol (entry 1), 3-methyl-1-cyclohexanol (entry 2), and 2-*tert*-butyl-1-cyclohexanol (entry 3) to be separated. Moreover, acetylation of 2-chloro-1-cyclohexanol (entry 6) and 4-*tert*-butyl-1-cyclohexanol (entry 7) increases the separation factor. 

When the acylation method was applied to polysubstituted cyclic alcohols, the results were not as good as previous ones. Now, only acetylation of *D,L*-menthol produced an increase in the separation factor value of the two enantiomers. Both stereoisomers have been already acetylated by Jereb and coworkers using a similar method [31]. They have already demonstrated that any isomerization was provoked in both compounds when acetylated separately. The *α* value was higher than the *α* obtained in some *α*, *β*, and *γ* permethylated cyclodextrins [13, 33] and in stationary GC phases of other cyclodextrin derivatives [39–41]. 

Finally the acylation method was applied to three diols: 1,2-octanediol, 1,2-cyclohexanediol, and 1,3-cyclohexanediol (Table 6). Now, one or both hydroxyl groups can be acetylated. In order to determine the retention time of the mono and diacetylated stereoisomers, two acylations reactions were carried out, one with a 1 : 4 molar ratio alcohol : acid and the other with a 1 : 1.5 molar ratio. When the molar ratio alcohol : acid was 1 : 1.5, there were more molecules with only one hydroxyl group acetylated than with the two hydroxyl groups acetylated (Figures 3, 4 and 5). However, when the molar ratio was 1 : 4, the numbers of diacetylated molecules were higher than the monoacetylated molecules. The samples were also analysed by NMR and the ^1^H and ^13^C spectra were in concordance with the chromatograms. The acetylation of one hydroxyl group increased the *α* value, compared with their corresponding alcohols; moreover, the *α* value was higher when both hydroxyl groups were acetylated. Thus, the diacetylation of 1,2-octanediol produced an increase in the separation factor value of the two enantiomers (1.03 for the diol and 1.34 for the diacetylated derivative). 

Li et al. obtained a similar *α* value (1.05) for the 1,2-octanediol on a **β**-2,6-di-*O*-pentyl-3-*O*-trifluoroacetylated cyclodextrin but lower than the separation of mono- and diacetates in our column [43]. The acetylation of *trans*-1,3-cyclohexanediol allowed the separation of its stereoisomers and the two sets of 1,2-cyclohexanediol stereoisomers were separated when acetylation of one of the hydroxyl groups took place. The *cis*-1,3-cyclohexanediol is a *meso*-form. However, when one of the hydroxyl groups was acetylated one chiral carbon was formed and the enantiomers could be separated in the column. When both hydroxyls groups were acetylated, the compound was again a *meso*-form. Finally, we would like to point out the simple handling of the reaction carried out prior to the GC analysis. Any further treatment is not needed once the reaction is finished but dilution and filtration of the reaction crude. Any loss of resolution capacity has not been observed in the chromatographic column after carrying out all of the described studies. 

## 4. Conclusions

We have demonstrated that the present procedure with iodine/carboxylic acid without adding solvent provides a very efficient method of esterification of numerous acyclic and cyclic chiral alcohols. This procedure allows a fast analysis of these compounds by chiral-phase GC. The main advantages of this method are its operational simplicity, the ready availability, and nontoxic nature of the reagent, and its general applicability. Near equimolar amounts of alcohol and carboxylic acid are typically used, thus avoiding waste and providing very simple experimental and workup procedures. Furthermore, acetylation of acyclic and cyclic alcohols usually increases the separation factor of the isomers.

## Figures and Tables

**Table 1:** Influence of solvent presence, reaction time, and molar ratio 2-heptanol : acetic acid in the 1-methylhexyl acetate yields.

Entry	Solvent	Time (h)	Molar ratio (2-heptanol : acetic acid)	% 1-methylhexyl acetate	SD	Tukey-Kramer*
1	No solvent	24	1 : 2	90.63	0.51	A
2		24	1 : 6	90.03	2.12	A B
3		24	1 : 10	84.00	1.79	B
4		48	1 : 2	91.76	2.84	A
5		48	1 : 6	93.74	2.93	A
6		48	1 : 10	93.11	1.26	A

7	*tert*-Butanol	24	1 : 2	16.63	1.05	C
8		24	1 : 6	34.67	0.04	D
9		24	1 : 10	56.20	0.01	E
10		48	1 : 2	81.72	1.17	B
11		48	1 : 6	88.00	0.09	A B
12		48	1 : 10	82.11	3.22	B

*Tukey-Kramer pairwise differences adjustment method, *n* = 3.

**Table 2:** Influence of reaction time and molar ratios 2-heptanol : acetic acid in the 1-methylhexyl acetate yields.

Entry	Molar ratio (heptanol : acetic acid)	Time (h)	% 1-methylhexyl acetate	SD	Tukey-Kramer*
1	1 : 1	24	71.04	—	A
2		48	87.03	1.70	B

3	1 : 1.5	4	76.60	0.97	C A
4		24	82.42	3.61	D B
5		48	91.48	3.16	E B

6	1 : 2	4	65.49	5.11	F A
7		8	78.58	0.82	A C D
8		24	90.63	0.51	G B
9		48	91.76	2.84	G B

*Tukey-Kramer pairwise differences adjustment method, *n* = 3.

**Table 3:** Effect of the temperature on the synthesis of 1-methylhexyl acetate. Reaction conditions: 1 : 1 molar ratio acetic acid : alcohol, 24 h and no solvent.

Entry	Temperature (°C)	% 1-methylhexyl acetate	SD	Tukey-Kramer*
1	100	71.24	2.15	A
2	120	64.13	1.11	B
3	140	66.41	2.56	B

*Tukey-Kramer pairwise differences adjustment method, = 3.

**Table 4:** Separation factor values (*α*) of the enantiomers of some acyclic alcohols and their corresponding acetyl derivatives.

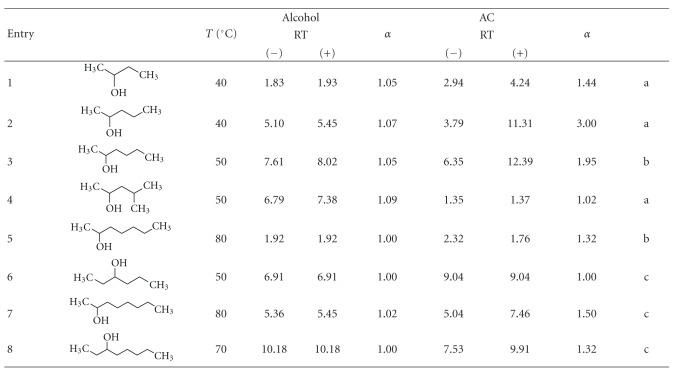

*T*: oven GC temperature (°C); RT: retention time; *α*: separation factor; AC: acetyl derivatives; the elution order of the acetylated enantiomers was assumed from the literature [34, 35]; a: ^1^H and ^13^C NMR spectral data were in agreement with those published in [37]; b: ^1^H and ^13^C NMR spectral data were in agreement with those published in [38]; c: ^1^H and ^13^C NMR spectral data in the appendix.

**Table 5:** Separation factor values (*α*) of the stereoisomers of some cyclic alcohols and their corresponding acetyl derivatives.

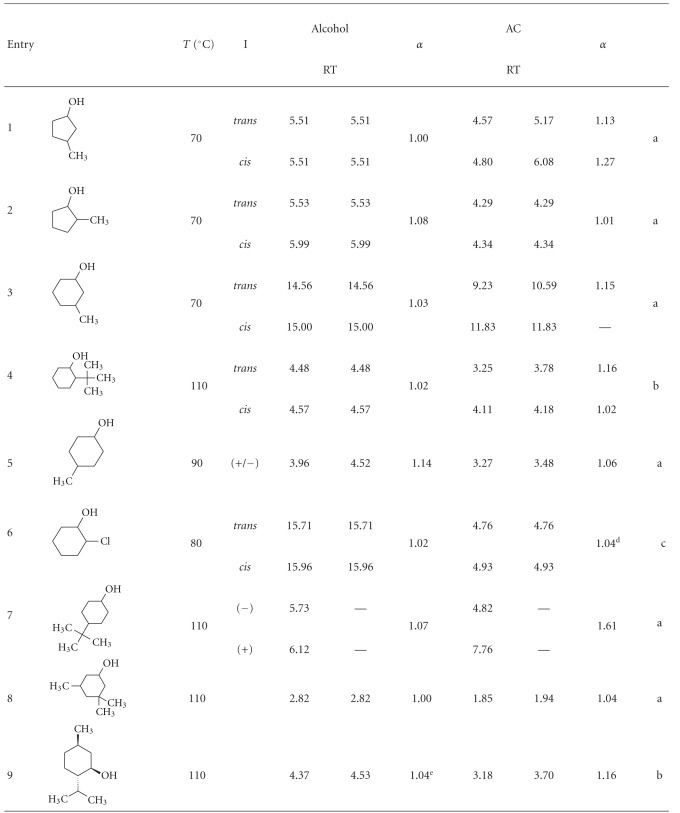

*T*: GC analysis temperature, (°C); I: isomer; RT: retention time; AC: acetyl derivatives; the elution order of the acetylated enantiomers was assumed from the literature [34, 35]; a: ^1^H and ^13^C NMR spectral data in the appendix; b: ^1^H and ^13^C NMR spectral data were in agreement with those published in [38]; c: ^1^H and ^13^C NMR spectral data were in agreement with those published in [42]; d: 1 : 10 ratio alcohol : acid; e: reversal of the elution order.

**Table 6:** Separation factor values (*α*) of the stereoisomers of some diols and their corresponding acetyl derivatives.

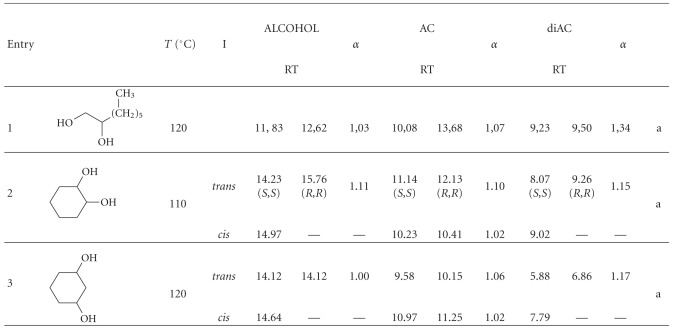

*T*: GC analysis temperature, (°C); I: isomer; RT: retention time; AC: monoacetyl derivative; diAC: diacetyl derivative; the elution order of the acetylated enantiomers was assumed from the literature [34, 35]; a: ^1^H and ^13^C NMR spectral data in the appendix.

## Appendix

### NMR Data of the Acetylated Alcohols, Analysed by a Varian 400, Solvent Acetone-d

1-Ethylbutyl Acetate
^1^H NMR (400 MHz, acetone) *δ* 4.81–4.72 (m, 1H), 1.99 (s, 3H), 1.63–1.44 (m, 4H), 1.41–1.22 (m, 2H), 0.89 (t, *J* = 7.47, Hz 3H), 0.85 (t, *J* = 7.47 Hz, 3H). ^13^C NMR (101 MHz, acetone) *δ* 170.08, 74.31, 35.71, 26.85, 20.14, 18.35, 13.33, 9.02.

1-Methylheptyl Acetate
^1^H NMR (400 MHz, acetone) *δ* 4.89–4.76 (m, 1H), 1.96 (s, 3H), 1.60–1.43 (m, 2H), 1.28 (m, 8H), 1.16 (d, *J* = 6.27 Hz, 3H), 0.88 (t, *J* = 6.8 Hz, 3H). ^13^C NMR (101 MHz, acetone) *δ* 169.65, 70.17, 35.73, 31.59, 29.15, 25.19, 22.34, 20.28, 19.35, 13.43.

1-Ethylhexyl Acetate
^1^H NMR (400 MHz, acetone) *δ* 4.76 (dt, *J* = 7.2, 6.2 Hz, 1H), 1.98 (s, 3H), 1.63–1.46 (m, 4H), 1.37–1.21 (m, 6H), 0.88 (t, *J* = 7,46 Hz, 3H), 0.86 (t, *J* = 7, 46 Hz, 3H). ^13^C NMR (101 MHz, acetone) *δ* 169.86, 74.64, 33.46, 31.52, 26.80, 24.83, 22.30, 20.14, 13.37, 9.01.

cis-3-Methylcyclopentyl Acetate
^1^H NMR (400 MHz, acetone) *δ* 3.90 (m, 1H), 1.93 (s, 3H), 1.98 (m, 1H), 1.68 (m, 1H), 1.59–1.49 (m, 3H), 1.29 (m, 1H), 1.16 (m, 1H), 0.98 (d, 3H). ^13^C NMR (101 MHz, acetone) *δ* 169.70, 76.14, 41.16, 32.76, 32.36, 32.30, 20.27 CH3, 20.24.

trans-3-Methylcyclopentyl Acetate
^1^H NMR (400 MHz, acetone) *δ* 5.07 (m, 1H), 1.94 (s, 3H), 1.83–1.72 (m, 2H), 1.64–1.53 (m, 2H), 1.43–1.30 (m, 2H), 1.22–1.11 (m, 1H), 1.02 (dd, *J* = 5.3, 4.0 Hz, 3H). ^13^C NMR (101 MHz, acetone) *δ* 169.75, 76.16, 40.78, 32.84, 32.38, 32.33, 20.11, 19.52.

trans-2-Methylcyclopentyl Acetate
^1^H NMR (400 MHz, acetone) *δ* 4.62 (dt, *J* = 6.8, 4.6 Hz, 1H), 1.96 (m, 1H), 1.96 (s, 3H), 1.71–1.61 (m, 3H), 1.59–1.49 (m, 2H), 1.20 (m, 1H), 0.97 (d, *J* = 6.9 Hz, 3H). ^13^C NMR (101 MHz, acetone) *δ* 169.94, 82.07, 39.86, 31.56, 31.04, 22.06, 20.21, 17.53.

cis-2-Methylcyclopentyl Acetate
^1^H NMR (400 MHz, acetone) *δ* 4.81 (m, 1H), 1.97 (m, 1H), 1.97 (s, 3H), 1.68–1.57 (m, 3H), 1.53–1.43(m, 2H), 1.17 (m, 1H), 0.96 (d, *J* = 6.9 Hz, 3H). ^13^C NMR (101 MHz, acetone) *δ* 169.92, 80.21, 39.38, 32.43, 332.17, 22.22, 20.19, 17.52.

cis-3-Methylcyclopentyl Acetate
^1^H NMR (400 MHz, acetone) *δ* 4.98 (m, 1H), 1.98 (s, 3H), 1.93–1.86 (m, 2H), 1.65–1.35 (m, 2H), 1.30–1.13 (m, 2H), 1.02 (m, 1H), 0.86 (d, *J* = 6.6 Hz, 3H), 0.87 (m, 2H). ^13^C NMR (101 MHz, acetone) *δ* 169.44, 69.51, 38.12, 33.89, 31.20, 30.51, 26.87, 21.54, 20.40.

trans-3-Methylcyclopentyl Acetate
^1^H NMR (400 MHz, acetone) *δ* 4.61 (tt, *J* = 11.3, 4.3 Hz, 1H), 1.94 (m, 3H), 1.93–1.87 (m, 2H), 1.68–1.12 (m, 5H), 0.91 (d, *J* = 6.6 Hz, 3H), 0.89 (m, 2H). ^13^C NMR (101 MHz, acetone) *δ* 169.42, 72.49, 40.36, 35.36, 33.80, 31.13, 23.69, 21.68, 20.32.

trans-4-Methylcyclohexyl Acetate
^1^H NMR (400 MHz, acetone) *δ* 4.57 (tt, *J* = 11.2, 4.4 Hz, 1H), 1.94 (s, 3H), 1.77–1.66 (m, 2H), 1.45–1.24 (m, 5H), 1.10–0.99 (m, 2H), 0.88 (d, *J* = 6.5 Hz, 3H). ^13^C NMR (101 MHz, acetone) *δ* 169.49, 72.65, 32.48, 31.52, 31.45, 21.25, 20.33.

cis-4-Methylcyclohexyl Acetate
^1^H NMR (400 MHz, acetone) *δ* 4.89 (s, 1H), 1.98 (s, 3H), 1.92–1.84 (m, 2H), 1.82–1.68 (m, 2H), 1.52 (m, 3H), 0.95 (m, 2H), 0.90 (d, *J* = 6.4 Hz, 3H). ^13^C NMR (101 MHz, acetone) *δ* 169.45, 69.01, 32.22, 31.52, 31.14, 21.25, 21.45.

cis-4-tert-Butylcyclohexyl Acetate
^1^H NMR (400 MHz, acetone) *δ* 4.55 (m, 1H), 1.94 (s, 3H), 1.81 (m, 2H), 1.36–0.98 (m, 7H), 0.86 (s, 9H). ^13^C NMR (101 MHz, acetone) *δ* 169.48, 72.97, 68.63, 47.03, 36.06, 31.95, 27.00, 20.32.

trans-4-tert-Butylcyclohexyl Acetate
^1^H NMR (400 MHz, acetone) *δ* 4.92 (m, 1H), 1.99 (s, 3H), 1.89 (m, 4H), 1.54–1.40 (m, 2H), 1.20–0.98 (m, 3H), 0.86 (s, 9H). ^13^C NMR (101 MHz, acetone) *δ* 169.45, 72.97, 69.95, 47.42, 36.01, 31.89, 26.86, 20.82.

cis-3,3,5-Trimethylcyclohexyl Acetate
^1^H NMR (400 MHz, acetone) *δ* 4.83 (tt, *J* = 11.6, 4.4 Hz, 1H), 1.94 (s, 3H), 1.93–1.89 (m, 1H), 1.78–1.62 (m, 1H), 1.35 (ddt, *J* = 13.1, 3.8, 2.0 Hz, 2H), 1.08 (m, 2H), 0.94 (s, 6H), 0.90 (d, *J* = 6.5 Hz, 3H), 0.96–0.78 (m, 1H). ^13^C NMR (101 MHz, acetone) *δ* 169.60, 70.21, 47.23, 43.82, 40.25, 32.45, 31.79, 26.91, 24.88, 21.74, 20.37.

trans-3,3,5-Trimethylcyclohexyl Acetate
^1^H NMR (400 MHz, acetone) *δ* 5.02 (s, 1H), 1.96 (s, 3H), 1.90 (m, 1H), 1.78–1.61 (m, 2H), 1.47–1.39 (m, 1H), 1.06–0.77 (m, 3H), 0.89 (d, *J* = 6.5 Hz, 3H), 0.88 (s, 6H). ^13^C NMR (101 MHz, acetone) *δ* 169.34, 70.17, 47.81, 40.87, 38.08, 33.38, 30.26, 26.76, 23.22, 21.93, 20.49.

2-Hydroxyoctyl Acetate
^1^H NMR (400 MHz, acetone) *δ* 4.01–3.88 (m, 2H), 3.43 (m, 1H), 2.00 (s, 3H), 1.52–1.18 (m, 10H), 0.88 (t, *J* = 7.16 Hz 3H). ^13^C NMR (101 MHz, acetone) *δ* 170.12, 71.14, 69.12, 33.56, 31.68, 29.16, 25.08, 22.35, 19.88, 13.44.

1-(Hydroxymethyl)heptyl Acetate
^1^H NMR (400 MHz, acetone) *δ* 4.85 (m, 1H), 3.55 (m, 2H), 1.99 (s, 3H), 1.65–1.20 (m, 10H), 0.88 (t, *J* = 7.16 Hz 3H). ^13^C NMR (101 MHz, acetone) *δ* 169.90, 75.81, 68.34, 31.56, 30.47, 29.03, 25.63, 22.38, 20.20, 13.45.

1-[(Acetyloxy)methyl]heptyl Acetate
^1^H NMR (400 MHz, acetone) *δ* 5.01(m, 1H), 4.21 (dd, *J* = 11.9, 3.4 Hz, 1H), 4.5-3,93 (m, 1H), 1.99 (s, 3H), 1.98 (s, 3H), 1.65–1.20 (m, 10 H), 0.88 (t, *J* = 7.16 Hz 3H). ^13^C NMR (101 MHz, acetone) *δ* 169.98, 169.79, 74.67, 68.66, 31.62, 30.40, 28.84, 24.90, 22.31, 20.03, 19.73, 13.41.

cis-2-Hydroxycyclohexyl Acetate
^1^H NMR (400 MHz, acetone) *δ* 4.82 (dd, *J* = 7.1, 4.0 Hz, 1H), 4.37 (s, OH), 4.04 (m, 1H), 1.99 (s, 3H), 1.82 (m, 1H), 1.77–1.61 (m, 4H), 1.31 (m, 2H), 1.41–1.21 (m, 1H). ^13^C NMR (101 MHz, acetone) *δ* 169.76, 73.31, 67.99, 29.54, 28.08, 21.90, 21.15, 20.62.

cis-2-(Acetyloxy)cyclohexyl Acetate
^1^H NMR (400 MHz, acetone) *δ* 4.96 (d, *J* = 8.4 Hz, 2H), 1.97 (s, 6H), 1.80 (m, 2H), 1.69–1.59 (m, 2H), 1.53–1.40 (m, 4H). ^13^C NMR (101 MHz, acetone) *δ* 169.49, 73.78, 29.54, 27.30, 20.09.

trans-2-Hydroxycyclohexyl Acetate
^1^H NMR (400 MHz, acetone) *δ* 4.53 (d, *J* = 8.9 Hz, 1H), 3.49 (m, 1H), 3.30 (s, OH), 1.97 (s, 3H), 1.91 (m, 2H), 1.76–1.59 (m, 2H), 1.39–1.16 (m, 4H). ^13^C NMR (101 MHz, acetone) *δ* 169.45, 77.15, 71.09, 32.88, 23.60, 3.04, 20.09, 19.64.

trans-2-(Acetyloxy)cyclohexyl Acetate
^1^H NMR (400 MHz, acetone) *δ* 4.72 (m, 2H), 1.95 (s, 6H), 1.92 (m, 2H), 1.64 (m, 2H), 1.243–1.23(m, 4H). ^13^C NMR (101 MHz, acetone) *δ* 169.97, 73.05, 29.66, 23.44, 20.10.

cis-3-Hydroxycyclohexyl Acetate
^1^H NMR (400 MHz, acetone) *δ* 4.62 (m, 1H), 3.57 (m, 1H), 4.86 (s, OH), 1.95 (s, 3H), 1.93–1.77 (m, 1H), 1.77–1.61 (m, 1H), 1.61–1.46 (m, 2H), 1.45–1.31 (m, 3H), 1.31–1.06 (m, 1H). ^13^C NMR (101 MHz, acetone) *δ* 169.34, 70.89, 65.75, 38.70, 36.92, 30.27, 20.21, 18.86.

trans-3-Hydroxycyclohexyl Acetate
^1^H NMR (400 MHz, acetone) *δ* 5.01 (m, 1H), 3.96 (m, 1H), 4.86 (s, OH), 1.98 (s, 3H), 1.93–1.77 (m, 1H), 1.77–1.61 (m, 1H), 1.61–1.46 (m, 2H), 1.45–1.31 (m, 3H), 1.31–1.06 (m, 1H). ^13^C NMR (101 MHz, acetone) *δ* 169.43 C, 69.93, 65.58, 41.29, 35.20, 29.82, 20.29, 19.00.

cis-3-(Acetyloxy)cyclohexyl Acetate
^1^H NMR (400 MHz, acetone) *δ* 4.69 (m, 2H), 1.96 (s, 6H), 1.94–1.78 (m, 3H), 1.78–1.61 (m, 1H), 1.61–1.31 (m, 2H), 1.31–1.07 (m, 2H). ^13^C NMR (101 MHz, acetone) *δ* 171.09, 70.05, 34.50, 30.87, 20.38, 19.78.

trans-3-(Acetyloxy)cyclohexyl Acetate
^1^H NMR (400 MHz, acetone) *δ* 5.06 (m, 2H), 1.96 (s, 6H), 1.94–1.78 (m, 3H), 1.78–1.61 (m, 1H), 1.61–1.31 (m, 2H), 1.31–1.07 (m, 2H). ^13^C NMR (101 MHz, acetone) *δ* 171.09, 69.45, 34.57, 30.47, 20.33, 19.58.
